# Root ABA Accumulation Enhances Rice Seedling Drought Tolerance under Ammonium Supply: Interaction with Aquaporins

**DOI:** 10.3389/fpls.2016.01206

**Published:** 2016-08-10

**Authors:** Lei Ding, Yingrui Li, Ying Wang, Limin Gao, Min Wang, François Chaumont, Qirong Shen, Shiwei Guo

**Affiliations:** ^1^Jiangsu Key Lab for Organic Waste Utilization, Nanjing Agricultural UniversityNanjing, China; ^2^Institut des Sciences de la Vie, Université catholique de LouvainLouvain-la-Neuve, Belgium

**Keywords:** rice, water uptake, ABA, aquaporin, drought stress

## Abstract

In previous studies, we demonstrated that ammonium nutrition enhances the drought tolerance of rice seedlings compared to nitrate nutrition and contributes to a higher root water uptake ability. It remains unclear why rice seedlings maintain a higher water uptake ability when supplied with ammonium under drought stress. Here, we focused on the effects of nitrogen form and drought stress on root *abscisic acid* (ABA) concentration and aquaporin expression using hydroponics experiments and stimulating drought stress with 10% PEG6000. Drought stress decreased the leaf photosynthetic rate and stomatal conductivity and increased the leaf temperature of plants supplied with either ammonium or nitrate, but especially under nitrate supply. After 4 h of PEG treatment, the root protoplast water permeability and the expression of root *PIP* and *TIP* genes decreased in plants supplied with ammonium or nitrate. After 24 h of PEG treatment, the root hydraulic conductivity, the protoplast water permeability, and the expression of some aquaporin genes increased in plants supplied with ammonium compared to those under non-PEG treatment. Root ABA accumulation was induced by 24 h of PEG treatment, especially in plants supplied with ammonium. The addition of exogenous ABA decreased the expression of PIP and TIP genes under non-PEG treatment but increased the expression of some of them under PEG treatment. We concluded that drought stress induced a down-regulation of aquaporin expression, which appeared earlier than did root ABA accumulation. With continued drought stress, aquaporin expression and activity increased due to root ABA accumulation in plants supplied with ammonium.

## Introduction

Previous studies demonstrated that ammonium nitrogen (NH_4_^+^) enhances rice seedling drought tolerance due to a higher root water uptake ability (Gao et al., 2010; Yang et al., 2012) compared to that of seedlings under nitrate nitrogen (NO_3_^-^) supply. Under drought stress, Yang et al. (2012) reported decreased root hydraulic conductivity in rice plants supplied with nitrate, illustrated by the down-regulation of aquaporin activity and the increased formation of root cortical aerenchyma. It was found that short-term simulated drought stress could increase root AQP expression, activity and root hydraulic conductivity under NH_4_^+^ supply but not under nitrate supply (Ding et al., 2015).

Abscisic acid (ABA) potentially plays important roles in AQP regulation and root water uptake in plants facing different nitrogen forms and/or drought stress (Schraut et al., 2005; Parent et al., 2009). In most studies, application of exogenous ABA increased the root *PIP* gene expression under normal water conditions (Jang et al., 2004; Zhu et al., 2005; Lian et al., 2006). Under drought stress, root ABA accumulation was indispensable for regulating *AQP* expression (Kaldenhoff et al., 2008; Parent et al., 2009) and for enhancing plant growth (Sharp, 2002; Zhang et al., 2006). Actually, in these conditions, roots-perceived water deficit and accumulated ABA, which would be transported to the leaves to regulate stomatal closure (Zhang and Davies, 1990; Davies and Zhang, 1991; Sengupta et al., 2011). A positive correlation has been observed between drought stress and root ABA accumulation in beans (Puertolas et al., 2013), potato (Puertolas et al., 2015), maize (Zhang and Davies, 1989), rice and *Arabidopsis* (Xu et al., 2013). Using ‘one shoot-two roots’ potato under partial root-zone drying (PRD), Puertolas et al. (2015) also showed that ABA only accumulated in dry-side roots, which further illustrated this positive correlation. In addition, plant aerial parts accumulated ABA under drought stress in tomato (Perez-Perez and Dodd, 2015), wheat (Guóth et al., 2009), and hops (Korovetska et al., 2014).

However, it remains unclear how nitrogen form affects ABA dynamics, such as how changes in ABA amount regulate root AQP expression and water uptake in plants under drought stress. It was reported that root and aerial tissues accumulated more ABA when NH_4_^+^ is supplied as a sole nitrogen source in castor bean (Peuke et al., 1994), pea (Zdunek and Lips, 2001), and tomato (Rahayu et al., 2005). We hypothesized that rice roots could accumulate more ABA and further stimulate AQP expression under drought stress with NH_4_^+^ supply. In the present study, we aimed to determine (1) how nitrogen form and drought stress affect ABA dynamics and (2) the potential correlation between ABA change and AQP regulation in roots under drought stress.

## Materials and Methods

### Plant Material and Growth Conditions

Rice seeds (*Oryza sativa* L., cv. ‘Shanyou 63’ hybrid *indica* China) were disinfected in 10% H_2_O_2_ (W/W) for 30 min and then germinated in a plastic basket (25 cm × 18 cm) with mesh. After the seedlings had developed an average of 2.5 visible leaves, they were transplanted to a 7-L plastic box containing a quarter-strength mixture of NH_4_^+^ and NO_3_^-^ (ANN) nutrient solution (Ding et al., 2015). After 3 days, the rice seedlings were transferred to half-strength ANN for 5 days and then supplied with full-strength ANN for 1 week, after which the seedlings were supplied with either NH_4_^+^ (AN) or NO_3_^-^ (NN) nutrient solution. After an additional week, the seedlings were subjected to simulated drought stress by the addition of 10% PEG (10% w/v, MW 6000) to the nutrient solutions (-0.15 MPa). Four treatments were applied: AN, NN, NH_4_^+^ plus 10% PEG 6000 (ANP) and NO_3_^-^ plus 10% PEG 6000 (NNP). For exogenous ABA treatment, 5 μM ABA in nutrient solution was added.

Cucumber plants were cultured identically to rice plants with the same nutrient solution in 1-L plastic cup. For simulating drought stress, 2% (w/v) PEG6000 was added into the nutrient solution.

The temperature in the glasshouse was maintained at 30°C during the day and 18°C at night. Light was supplied by SON-T AGRO 400 W bulbs; the light intensity was maintained at a minimum of 1000 μmol photons m^-2^ s^-1^ (photosynthetically active radiation) at the leaf level using a 14-h photoperiod.

### Gas-Exchange Measurement and Thermo Imaging

After 24 h of treatment with PEG, the light-saturated photosynthesis of newly expanded leaves was measured from 09:00 to 11:00 using the Li-Cor 6400 portable photosynthesis system. The leaf temperature during measurement was maintained at 28°C, and the photosynthetic photon flux density (PPFD) was 1500 μmol m^-2^ s^-1^.

Meanwhile, infrared images were obtained using an infrared camera (SC620, FLIR Systems, Inc., USA) with a spectral sensitivity ranging from 7.5 to 13 mm and a spatial resolution of 0.65 mrad.

### Root Hydraulic Conductivity Measurement and Root Protoplast Swelling Analysis

After 24 h of treatment with PEG, root hydraulic conductance was measured using a high-pressure flow meter (HPFM; Decagon Devices, Pullman, WA, USA) according to Ding et al. (2015). Root protoplasts were isolated after 4 and 24 h of PEG treatment, and a swelling assay was conducted to analyze the water permeability coefficient P_os_ according to a previous method (Ding et al., 2015).

### RNA Isolation and Quantitative Real Time PCR (RT-qPCR)

Root samples were harvested after 4 and 24 h of PEG and ABA treatments, immediately frozen in liquid nitrogen, and then stored at -70°C until RNA isolation. The total RNA was extracted with TRIzol reagent (Invitrogen, USA) according to the manufacturer’s instructions. cDNA was synthesized using the PrimeScript^TM^ RT reagent Kit with gDNA Eraser (Takara, Dalian, China). Reverse transcription quantitative real time polymerase chain reaction (RT-qPCR) was performed using the ABI 7500 Real-Time PCR system, and the products were labeled using the SYBR Green master mix (SYBR^®^ Premix Ex Taq^TM^ II (Tli RNaseH Plus); TaKaRa, Dalian, China). The primers for RT-qPCR were according to Sakurai-Ishikawa et al. (2011), and the 18 sRNA was used as a housekeeping gene. Genes identifiers were listed in Supplementary Table S1. The relative gene expression was calculated with the 2^-ΔCt^ method.

### ABA Detection in Roots, Leaves, and Xylem Sap

Root samples were harvested after PEG treatment for 2, 4, 12 and 24 h, followed by storage at -70°C. Leaf samples were harvested after 24 h of PEG treatment. Both the roots and leaves were freeze dried and extracted in glass-distilled water using approximately 1.2 ml per 40 mg dry weight, boiled for 1–2 min, and shaken at 4°C overnight. The extracts were centrifuged, and the supernatants were assayed with an enzyme-linked immunosorbent assay (ELISA; Plant hormone ABA; ELISA Kit, CUSABIO, USA).

To detect xylem sap ABA, xylem sap was collected after 24 h of PEG treatment. The plants were de-topped approximately 2 cm above the interface of the roots and shoots, and the exudation was immediately cleaned with filter paper to avoid contamination. Absorbent cotton was placed on the top of each piece of de-topped xylem and covered with plastic film to avoid evaporation. Xylem sap was collected from the cotton with a syringe and then frozen at -20°C for the ABA assay. Frozen xylem sap was allowed to thaw for approximately 45 min before being assayed.

### Statistics

A one-way analysis of variance (ANOVA) was used to assess the differences in each parameter among the treatments using the JMP 9 statistical software package (SAS Institute, Cary, NC, USA). For gene expression analysis, R software package^1^ was used to generate a hierarchical cluster heat map and cluster tree. The significant differences (*P* < 0.05) among the treatments as determined by Student’s *t*-test are indicated with different letters.

## Results

### Effect of Nitrogen Form and Drought Stress on Leaf Gas Exchange and Temperature

**FIGURE 1:**
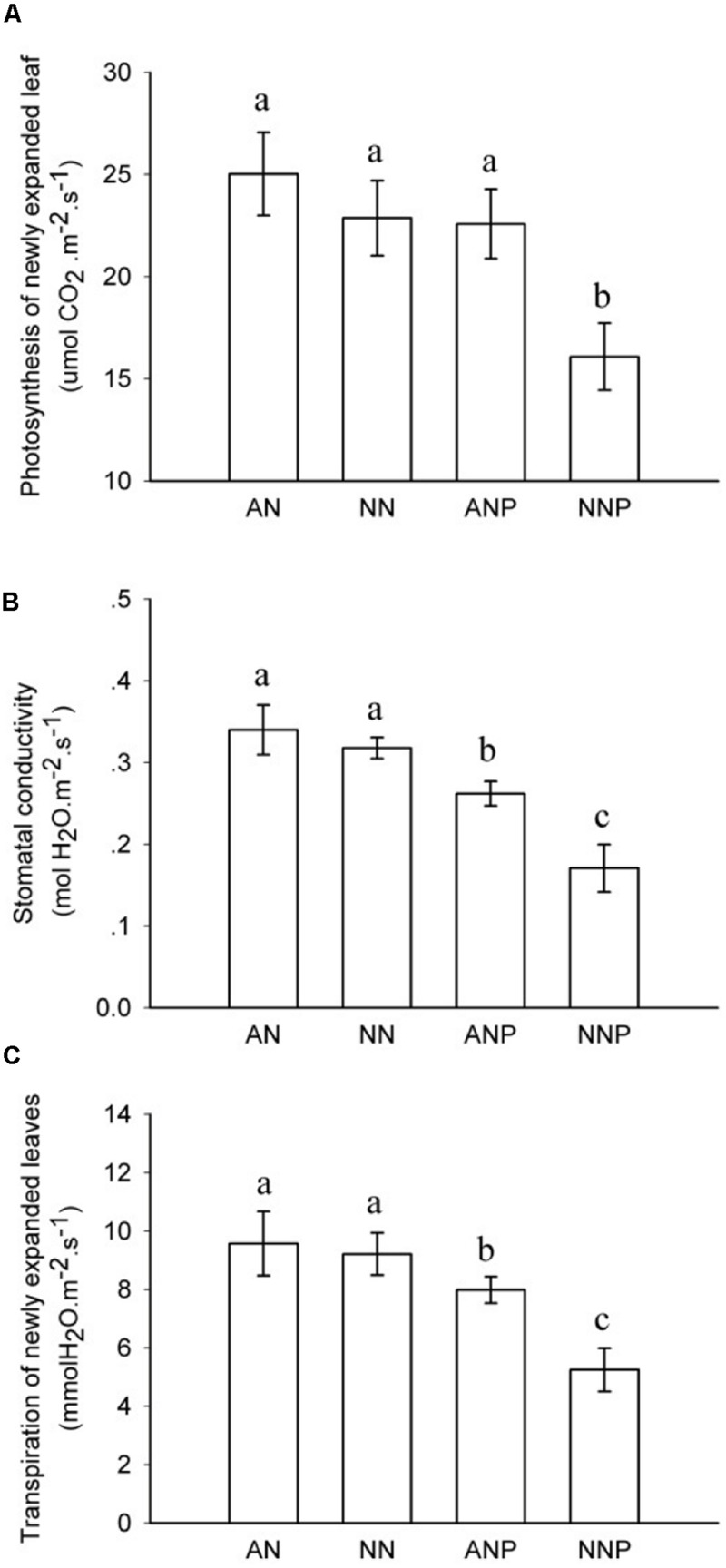
**Effects of different nitrogen forms and drought stress on newly expanded leaf photosynthetic rate **(A)**, stomatal conductivity **(B)**, and transpiration **(C)**.** The data represent the means of five replicates. The error bars indicate the ±SD. Significant differences (*P* < 0.05) between treatments are indicated by different letters. Rice seedlings were supplied with ammonium or nitrate under control and drought stress simulation by adding 10% PEG6000 (NH_4_^+^ + PEG as ANP; NO_3_^-^ + PEG as NNP). After 24 h of treatment with PEG, newly expanded leave were measured.

Under non-PEG treatment, no significant differences in the photosynthetic rate (Pn), stomatal conductivity (g_s_), or transpiration (Tr) were observed between NH_4_^+^- and NO_3_^-^-supplied plants (**Figure 1**). After 24 h of PEG treatment, g_s_ and Tr significantly decreased in plants supplied with either nitrogen form, and Pn decreased in NO_3_^-^-supplied plants. In plants supplied with NO_3_^-^, the g_s_ decreased from 0.32 to 0.17 mol m^-2^ s^-1^ and the Pn decreased by approximately 30% by PEG treatment from 22.9 to 16.1 μmol CO_2_ m^-2^ s^-1^ (**Figure 1**).

**FIGURE 2:**
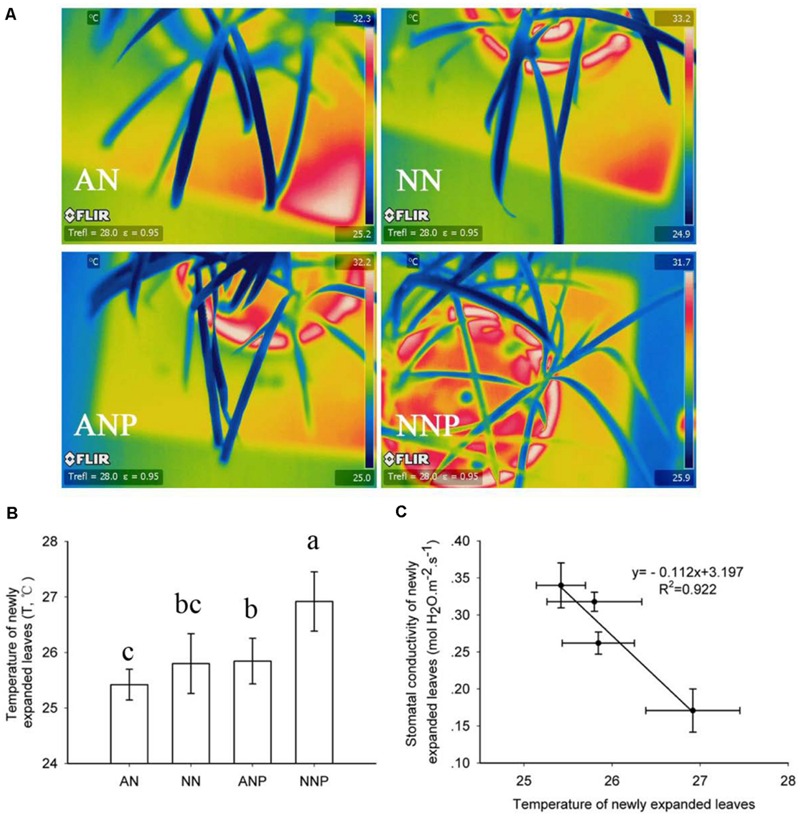
**Effects of different nitrogen forms and drought stress on leaf thermo image **(A)**, leaf temperature **(B)** and the correlation between stomatal conductivity and leaf temperature **(C)**.** A thermograph was taken before measuring the leaf photosynthetic rate. To calculate leaf temperature, 11 leaves were taken from the thermo image. The error bars indicate the ±SD. Significant differences (*P* < 0.05) between treatments are indicated by different letters. Rice seedlings were supplied with ammonium or nitrate under control and drought stress stimulation by adding 10% PEG6000 (NH_4_^+^ + PEG as ANP; NO_3_^-^ + PEG as NNP).

To investigate how drought stress affected leaf temperature, an infrared camera was used. A thermograph was used to determine the temperature difference. Leaves showed the highest temperature when supplied with NO_3_^-^ under PEG treatment (**Figures 2A,B**). There was a significant negative correlation between stomatal conductivity and leaf temperature (**Figure 2C**).

### Effect of Nitrogen Form and Drought Stress on Root Water Uptake Ability

**FIGURE 3:**
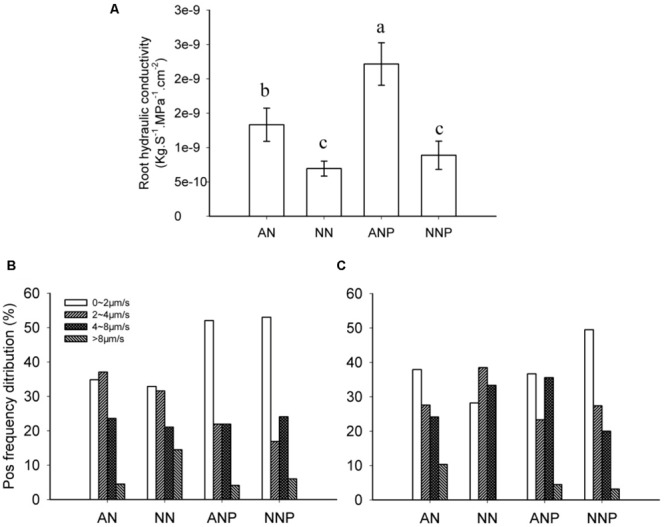
**Effects of different nitrogen forms and drought stress on root hydraulic conductivity **(A)** and the root protoplast water permeability coefficient P_os_**(B,C)**.** Root hydraulic conductivity was measured after 24 h of PEG treatment. Root protoplasts were isolated after 4 h **(B)** and 24 h **(C)** of PEG treatment. The data represent the means of four replicates. The error bars indicate the ±SD. Significant differences (*P* < 0.05) between treatments are indicated by different letters. Rice seedlings were supplied with ammonium or nitrate under control and drought stress stimulation by adding 10% PEG6000 (NH_4_^+^ + PEG as ANP; NO_3_^-^ + PEG as NNP).

After 24 h of PEG treatment, the root hydraulic conductivity increased significantly in plants supplied with NH_4_^+^. Compared to non-PEG treatment, drought stress enhanced the root hydraulic conductivity approximately twofold when the plants were fed NH_4_^+^ (**Figure 3A**). Similar results were not observed in plants supplied with NO_3_^-^.

To further investigate the effect of nitrogen form and drought stress on root cell water permeability, a root protoplast swelling assay was performed. After 4 h of PEG treatment, the water permeability P_os_ of protoplasts obtained from roots supplied with either nitrogen form decreased (**Figure 3B**). However, the Pos increased when the plants grew in presence of NH_4_^+^ supply after 24 h of PEG treatment compared with the P_os_ of cells coming from non-PEG treated plants; no change in P_os_ was observed when the plants were supplied with NO_3_^-^ (**Figure 3C**).

### Effect of Nitrogen Form and Drought Stress on Root *PIP* and *TIP* Gene Expression

**FIGURE 4:**
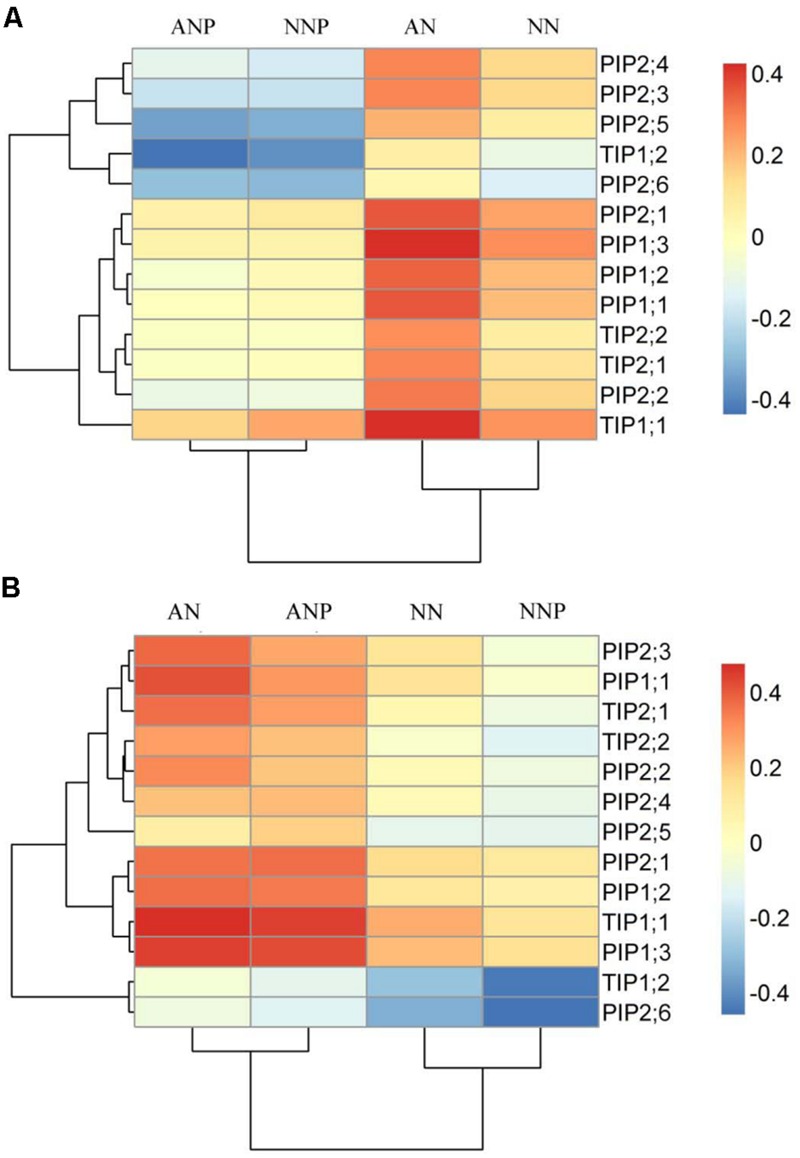
**Effects of different nitrogen forms and drought stress on root *PIP* and *TIP* gene expression.** Root *PIP* and *TIP* expression was detected after 4 h **(A)** and 24 h **(B)** of PEG treatment. The data were from RT-qPCR and were analyzed with 2^-ΔCt^ and further normalized with the log_10_ method. Red represents higher in transcript levels than blue colors as indicated in figure. The treatment tree and gene tree were generated based on the expression pattern. The data represent the means of three replicates. Rice seedlings were supplied with ammonium or nitrate under control and drought stress simulated by adding 10% PEG6000 (NH_4_^+^ + PEG as ANP; NO_3_^-^ + PEG as NNP).

To investigate how the expression of the aquaporin genes was affected in roots by drought stress, we measured the mRNA level of nine PIPs genes (PIP1;1 to PIP1;3, PIP2;1 to PIP2;6), including PIP1;1 to 1;3 and PIP2;1 to 2;6, and four TIPs genes (TIP1;1, TIP1;2, TIP2;1, and TIP2;2) by the RT-qPCR. Under non-PEG treatment, the expression of almost all the *PIP* and *TIP* genes was higher in plants supplied with NH_4_^+^ than that in those supplied with NO_3_^-^ (**Figure 4**). After 4 h of PEG treatment, the expression of all genes dramatically decreased in plants supplied with either nitrogen forms compared to that of non-PEG treatment, especially that of *PIP2;5, PIP2;6*, and TIP1;2. In contrast, after 24 h of PEG treatment, the expression of these genes showed different levels of increase compared to those under 4 h PEG treatment, especially in plants supplied with NH_4_^+^. Compared to non-PEG treatment, *PIP2;5* expression was higher under NH_4_^+^ supply after 24 h of PEG treatment (**Figure 4**).

### Effect of Nitrogen Form and Drought Stress on Root Endogenous Abscisic Acid (ABA), Xylem Sap ABA, and Leaf ABA

**FIGURE 5:**
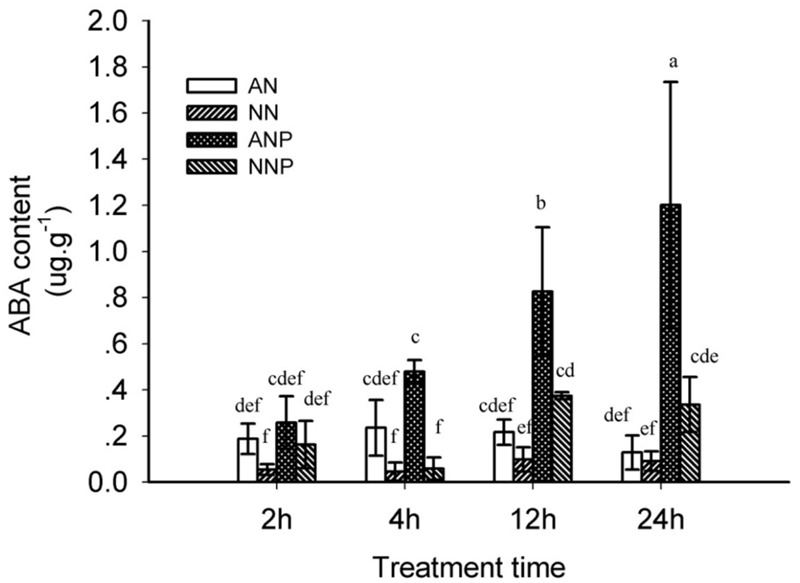
**Effects of different nitrogen forms and drought stress on the root ABA concentration.** Root ABA dynamics were detected after 2, 4, 12, and 24 h of PEG treatment. The data represent the means of three replicates. The error bars indicate the ±SD. Significant differences (*P* < 0.05) between treatments are indicated by different letters. Rice seedlings were supplied with ammonium (AN) or nitrate (NN) under control and drought stress simulation by adding 10% PEG6000 (NH_4_^+^ + PEG as ANP; NO_3_^-^ + PEG as NNP).

To investigate the potential relationship between aquaporin and ABA under drought stress, ABA concentration was measured after 2, 4, 12, and 24 h of PEG treatment. Under non-PEG treatment, the root ABA concentration was higher in plants supplied with NH_4_^+^ compared with the plants supplied with NO_3_^-^ (**Figure 5**). The ABA concentration was significantly higher in plants supplied with NH_4_^+^ after 12 h of PEG treatment than in plants under non-PEG treatment. In particular, after 24 h of treatment, the ABA concentration increased 10-fold from 0.13 to 1.2 μg g^-1^. In plants supplied with NO_3_^-^, PEG treatment increased the ABA concentration from 0.09 to 0.33 μg g^-1^. After 24 h of PEG treatment, the root ABA concentration was fourfold higher under NH_4_^+^ supply than that under NO_3_^-^ supply.

**FIGURE 6:**
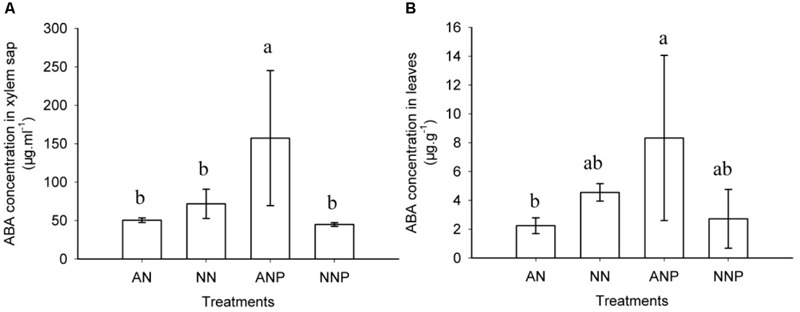
**Effects of different nitrogen forms and drought stress on xylem **(A)** and leaf **(B)** ABA concentration.** After 24 h of PEG treatment, xylem sap and leaves were harvested for ABA detection. The data represent the means of three replicates. The error bars indicate the ±SD. Significant differences (*P* < 0.05) between treatments are indicated by different letters. Rice seedlings were supplied with ammonium or nitrate under control and drought stress simulation by adding 10% PEG6000 (NH_4_^+^ + PEG as ANP; NO_3_^-^ + PEG as NNP).

In the aerial parts under non-PEG treatment, both xylem sap and leaf ABA concentrations were higher when supplied with NO_3_^-^, but not significantly different than supplied with NH_4_^+^ (**Figure 6A**). However, 24 h of PEG treatment significantly increased the xylem sap ABA concentration, which was threefold higher than that in non-PEG-treated plant supplied with NH_4_^+^. There was no increase in the xylem ABA concentration by PEG treatment in plants supplied with NO_3_^-^, which was significantly lower than that in plants supplied with NH_4_^+^ (**Figure 6A**). Compared to the xylem sap ABA change, the ABA concentration in the leaves showed the same tendency, increasing by PEG treatment from 2.24 to 8.33 μg g^-1^ in plants supplied with NH_4_^+^ (**Figure 6B**).

### Effect of Exogenous Abscisic Acid (ABA) on Root *AQP* Gene Expression

**FIGURE 7:**
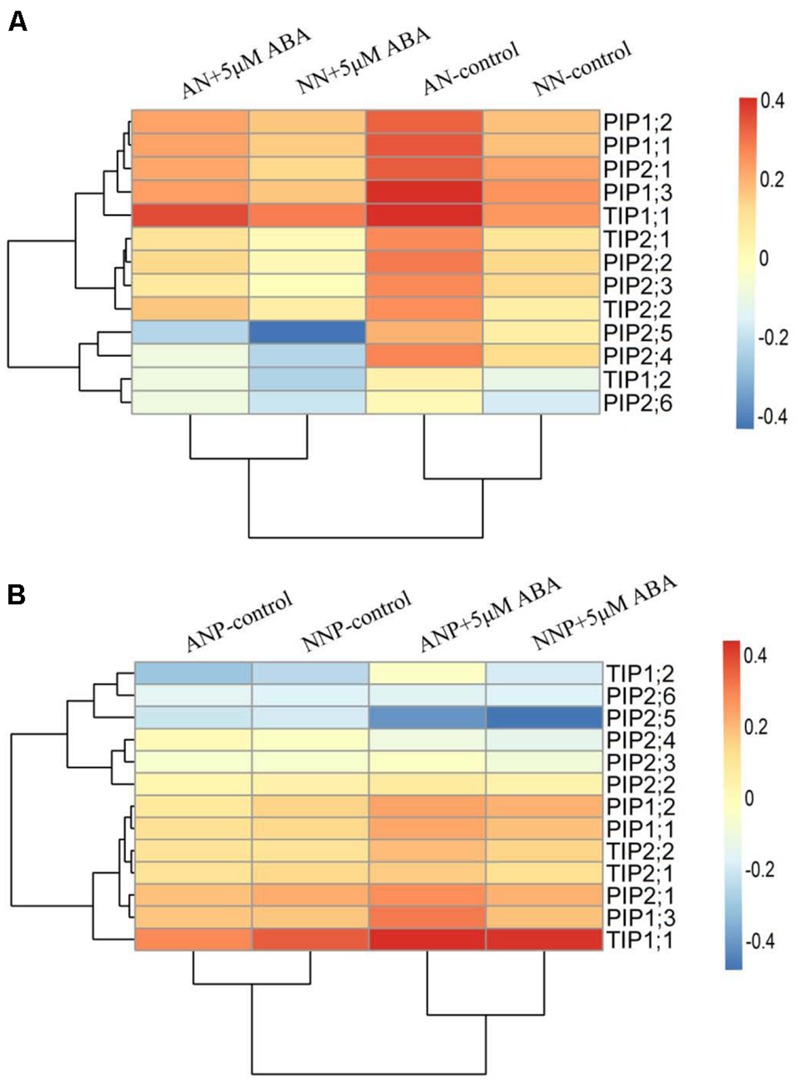
**Effects of exogenous ABA on root *PIP* and *TIP* gene expression under non-drought stress **(A)** and drought stress **(B)** conditions.** Exogenous ABA was applied to the nutrient solution, and the final concentration was 5 μM, as soon as drought stress was applied by using 10% PEG. After 4 h of ABA and/or drought stress treatment, root samples were harvested for gene expression analysis. The data were analyzed as in **Figure 4**. The treatments were ammonium (AN-control) and with ABA (AN + 5 μM ABA), nitrate (NN-control) and with ABA (NN + 5 μM ABA), ANP-control and ABA (ANP + 5 μM ABA), NNP-control and ABA (NNP + 5 μM ABA).

To further investigate the regulation by ABA of root *AQP* gene expression, exogenous ABA was applied to the roots. Under non-PEG treatment, exogenous ABA applied for 4 h decreased the expression of all PIP and TIP genes in plants supplied with either NH_4_^+^ or NO_3_^-^. Under PEG treatment, the application of exogenous ABA for 4 h increased *PIP1;1, PIP1;2, PIP1;3, PIP2;1, TIP1;1, TIP2;1*, and *TIP2;2* mRNA levels compared to that in non-ABA-applied plants (**Figure 7**) supplied with either NH_4_^+^ or NO_3_^-^, but especially under NH_4_^+^ supply. After 24 h of ABA treatment, no increase in gene expression was observed compared with non-ABA application under PEG treatment, and the expression of some genes decreased (Supplementary Figure S2).

## Discussion

### Effect of Drought Stress on Root Water Uptake

Drought stress increased the root hydraulic conductivity (Lpr) in plants supplied with ammonium (**Figure 3**). Numerous studies have reported Lpr was regulated by drought stress in different plant species, e.g., maize (Hachez et al., 2012), cucumber (Qian et al., 2015), tobacco (Mahdieh et al., 2008), grapevine (Vandeleur et al., 2009), and rice (Yang et al., 2012; Grondin et al., 2016). In these studies, regulation of the Lpr was shown to be dependent on the duration of the stress, the species and even the cultivars. First, long-term drought stress decreases Lpr compared to short-term stress, which might increase Lpr. Grondin et al. (2016) analyzed the Lpr of six different rice cultivars under long term drought stress, and five of them showed obvious conductivity decrease. Our previous results demonstrated no significant difference in the Lpr during several weeks of drought stress compared with normal water treatment in rice plants supplied with ammonium, while Lpr decreased in plants supplied with nitrate under the same conditions (Yang et al., 2012). It is possible that the roots Lpr decreased to avoid water depletion when the plant suffers long-term drought stress. Considering short-term PEG stress (24 h), Lpr increased in plants supplied with ammonium (**Figure 3**). Compared to our results, Hachez et al. (2012) reported that cell hydraulic conductivity increased approximately fourfold when maize plants stressed with 10% PEG for 2 h. Second, the Lpr varied according to the species under drought stress. Qian et al. (2015) reported that both root and cell hydraulic conductivity decreased in cucumber upon 140 mM PEG treatment for 2 and 24 h, while in tobacco, the root water uptake ability decreased upon PEG treatment (Ψ = -0.35 MPa) for 24 h (Mahdieh et al., 2008). In maize and rice plants, the hydraulic conductivity was increased by short-term drought stress. Both increased and decreased Lpr could enhance drought tolerance, representing different regulation strategies.

Increasing evidence indicates that aquaporin plays vital roles in the process of root radial water transport and affect Lpr, for review see (Chaumont and Tyerman, 2014). The contribution of aquaporin to Lpr is generally high, up to 79% under well-watered conditions and 85% under drought stress in rice plants (Grondin et al., 2016). In the present study, we investigated root protoplast water permeability and *PIP* and *TIP* genes expression in response to PEG treatment in presence of different nitrogen forms. We observed that 4 h of drought stress decreased root protoplast P_os_ in plants supplied with either ammonium or nitrate, while 24 h of drought stress increased P_os_ in plants supplied with ammonium (**Figures 3B,C**), which is consistent with previous result (Ding et al., 2015). Interestingly, this is in accordance with the decreased expression of all *PIP* and *TIP* genes observed upon 4 h of drought stress in plants supplied with either ammonium or nitrate and the dramatic increase in expression in plants supplied with ammonium after 24 h of stress (**Figure 4**).

In the present study, drought stress in presence of ammonium induced a decrease in *PIP2;5* gene expression after 4 h of treatment and an increase in its expression after 24 h (**Figure 4**). This flexible shift suggested that *PIP2;5* plays an important role in regulating radial water transport under drought stress. Sakurai-Ishikawa et al. (2011) reported that *OsPIP2;5* contributed significantly to water radial water movement during diurnal changes and mainly accumulated on the proximal end of the endodermis and in xylem parenchyma cells, where transport resistance is high. In maize plants, both 2 and 8 h of 10% PEG treatment increased *ZmPIP2;5* gene expression and protein content (Hachez et al., 2012). Considering *TIP* genes, Li et al. (2008) found them to be tightly related to tolerance to various stresses, including dehydration, salinity, ABA and seed germination in rice plants. *TIP1;2* and *TIP2;2* facilitated water transport when expressed in *Xenopus* oocytes. In addition, a 10 h 15% PEG treatment increased significantly *OsTIP1;1* expression, while in our study the expression of four *TIP* genes significantly increased after 24 h of PEG treatment compared to that after 4 h.

In conclusion, short-term 24 h PEG treatment increased root hydraulic conductivity, root protoplast water permeability and the expression of PIP and TIP genes, which might facilitate the water transport in and out the cells and the whole plant metabolism in plants supplied ammonium. However, after 4 h of PEG treatment, *AQP* expression and activity decreased to avoid cell dehydration in plants supplied with either ammonium or nitrate (**Figure 4**).

### The Interaction between ABA Change and Aquaporin Expression Regulation in Roots under Drought Stress

We observed that root ABA accumulation peaked after 24 h of PEG treatment in plants supplied with ammonium and increased by approximately 10-fold (**Figure 5**). In xylem sap and leaves, drought stress also induced ABA accumulation under ammonium supply (**Figure 6**). However, there is limited evidence suggesting how nitrogen form and drought stress affect the root ABA concentration. Our results showed that there was a greater ABA concentration in rice plants supplied with ammonium rather than with nitrate under drought stress, while no significant difference was observed under normal water condition (**Figure 5**). In wheat, there was no difference in root ABA concentration between plants supplied with ammonium and those supplied with nitrate (Chen et al., 1998). Root or aerial tissues accumulate more ABA when NH_4_^+^ supplied as a sole nitrogen source under non-drought stress in castor bean (Peuke et al., 1994), pea (Zdunek and Lips, 2001), and tomato (Rahayu et al., 2005). In cucumber, a higher but not significant root ABA concentration was observed when supplied with nitrate compared with the ammonium supply. Under drought stress, ABA change varied with duration of stress and nitrogen form (Supplementary Figure S1). It was therefore speculated that plant ABA is dynamically dependent on the nitrogen form, the species and the water status.

In roots, ABA plays important roles in regulating radial water transport and *AQP* gene expression, and in most cases, ABA increases root *AQP* gene expression and Lpr (Jang et al., 2004; Zhu et al., 2005; Mahdieh and Mostajeran, 2009; Parent et al., 2009). We observed an expression decrease in *PIP* and *TIP* genes by when the plants are incubated with 5 μM ABA under non-drought stress (**Figure 7A**; Supplementary Figure S2A). Consistent with this result, Beaudette et al. (2007) reported that 0.01 and 100 μM ABA incubation for 24 h decreased *PsPIP2;1* expression. However, they also demonstrated that 1 and 10 μM exogenous ABA increased *PsPIP2;1* expression. In maize plants, 1 μM ABA increased root cell hydraulic conductivity from 10 min to 1 h incubation, followed by a dramatic decrease after 2 h, indicating a negative effect of long term ABA treatments on the root water radial transport. Lian et al. (2006) reported that upland and lowland rice have different *PIP* expression levels affected by exogenous ABA apply, even though different PIP genes exhibited different regulation. Based on this evidence, it became controversial how ABA affects root *AQP* expression and hydraulic conductivity (Lpr), which depend on ABA dose, time responses and interaction with drought stress. However, the above results are based on exogenous ABA treatment. Regardless of the different results of exogenous ABA treatment, the endogenous ABA level should be more positively related to root *AQP* expression and Lpr regulation (Parent et al., 2009). These authors demonstrated that ABA is essential for root *AQP* expression by investigating ABA deficiency and over-accumulation maize plants; ABA-accumulated maize plants showed higher root *AQP* expression, while ABA deficient plants showed lower root *AQP* expression.

Under drought stress, 5 μM exogenous ABA induced an up-regulation of *PIP1;1, PIP1;2, PIP1;3, PIP2;1, TIP1;1, TIP2;1* and *TIP2;2* genes, especially in plants supplied with ammonium (**Figure 7B**). Moreover, drought stress induced more ABA accumulation in roots supplied with ammonium after 24 h of treatment, when root PIP and TIP expression was up-regulated (**Figure 5**). Our results showed that root ABA accumulation increase *AQP* expression induced by drought stress. Meanwhile, the *AQP* expression decreased by 4 h of drought stress independent of ABA regulation. In addition, we observed a higher ABA level, which could explain why the Pos was higher under drought stress in cucumber supplied with nitrate than in that supplied with ammonium (Supplementary Figure S1B). Despite the different effects of nitrogen form and drought stress on rice and cucumber root water uptake, ABA accumulation increased the root and/or cell water hydraulics under drought stress.

In aerial xylem sap and leaves, we observed that ABA levels increased when the plants are supplied with ammonium under drought stress (**Figure 6**), in which root ABA accumulated 10-fold after 24 h of drought stress (**Figure 5**). Under drought stress, the stomatal conductivity decreased in leaves supplied with either nitrogen form, especially in plants supplied with nitrate (**Figure 1**). In previous study, digital infrared thermography (DIT) was employed to detect changes in leaf temperature, which negative associated with leaf transpiration rate (Wang et al., 2012). However, the leaf transpiration was regulated by stomatal conductivity, and therefore thermo image could be an indicator for leaf stomatal opening (**Figure 2**).

Interestingly, there were higher levels of ABA and stomatal conductivity under drought stress in plants supplied with ammonium rather than with nitrate, indicating that ABA is indispensable for plants under drought stress. Two factors might simultaneously explain the higher stomatal conductivity and ABA under drought stress in plants supplied with ammonium rather than nitrate. First, drought stress could induce the alkalization of apoplastic sap; this process has a crosstalk with nitrate metabolism (Ehlert et al., 2011; Korovetska et al., 2014). Under low pH, most of the leaf ABA was stored in the symplast (inactivation), while drought stress increased the pH, activating ABA, which would reside in the apoplast and enter the stomata (Wilkinson et al., 2007). In the present study, more ABA was stored in the symplast under drought stress in plants supplied with ammonium; in contrast, as most of the ABA resides in the apoplast under nitrate supply, even less ABA could induce the stomatal closure. To support this, plants fertilized with nitrate will tend to show high xylem and apoplastic pH (Supplementary Figure S3; Mengel et al., 1994; Muhling and Lauchli, 2001). Second, ABA accumulation is essential for shoots growing under drought stress (Koornneef et al., 1998; Sharp, 2002). Under drought stress, ABA-deficient mutants wilt and die. As a result, a higher ABA concentration might induce partial stomatal closure, further reducing water loss under drought stress in plant supplied with ammonium. Consistently, Li et al. (2012) showed that drought stress decreased the leaf water potential under nitrate supply, further inducing chloroplast downsizing.

## Conclusion

**FIGURE 8:**
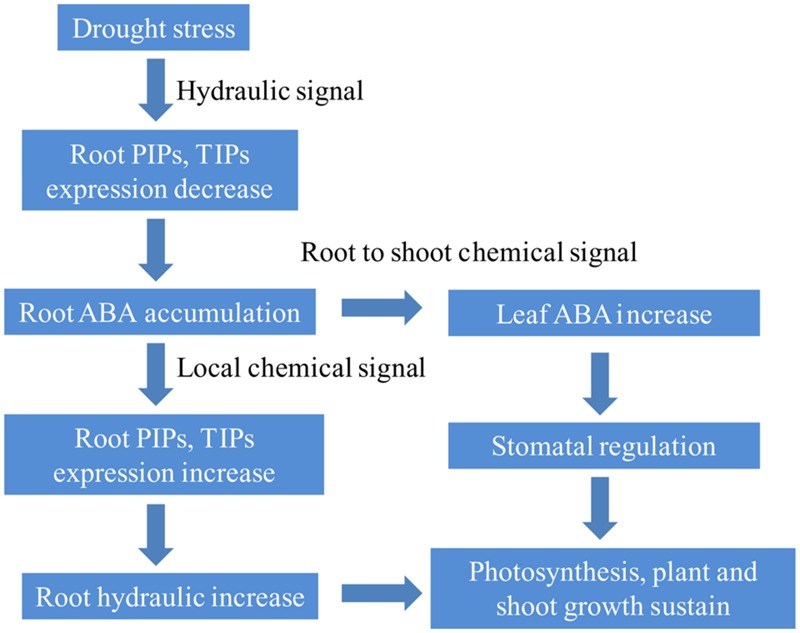
**The mechanism by which ammonium nutrition enhances rice seedling drought tolerance**.

Taken together, one of the possible mechanism of “ABA accumulation in enhanced seedling stage drought tolerance” is given in **Figure 8**. First, under drought stress, root *PIP* and *TIP* expression decreased immediately and intensely, while root ABA tended to accumulate. Second, root *PIP* and *TIP* expression increased with stress extension, resulting from endogenous ABA accumulation and further increasing root hydraulic conductivity. Third, root ABA accumulation induced aboveground ABA level increase, including in the leaves, as a result of stomatal closure (partially closure). Photosynthetic CO_2_ assimilation was maintained under drought stress in rice plants supplied with ammonium.

## Author Contributions

SG and LD designed the experiment. LD, YL, YW, and LG performed the experiments. LD analyzed the data. LD, MW, FC, QS, and SG wrote the paper.

## Conflict of Interest Statement

The authors declare that the research was conducted in the absence of any commercial or financial relationships that could be construed as a potential conflict of interest.

## Footnotes

1 http://www.r-project.org/

## Abbreviations

AN: ammonium
ANP: ammonium with PEG
AQP: aquaporin
NN: nitrate
NNP: nitrate with PEG
PEG: polyethylene glycol
PIP: plasma membrane intrinsic protein
TIP: tonoplast membrane intrinsic protein
