# Supplementary material for: Root ABA Accumulation Enhances Rice Seedling Drought Tolerance under Ammonium Supply: Interaction with Aquaporins
Source: Front Plant Sci. 2016 Aug 10;7:1206. doi: 10.3389/fpls.2016.01206 (PMC4979525; doi:10.3389/fpls.2016.01206)
Supplement: Supplementary file 1 [file Data_Sheet_1.DOCX]

Table S1 gene identifiers of all AQP used in this experiment

| **Gene** | **RAP id** |
| --- | --- |
| *OsPIP1;1* | Os02g0666200 |
| *OsPIP1;2* | Os04g0559700 |
| *OsPIP1;3* | Os02g0823100 |
| *OsPIP2;1* | Os07g0448800 |
| *OsPIP2;2* | Os02g0629200 |
| *OsPIP2;3* | Os04g0521100 |
| *OsPIP2;4* | Os07g0448100 |
| *OsPIP2;5* | Os07g0448400 |
| *OsPIP2;6* | Os04g0233400 |
| *OsTIP1;1* | Os03g0146100 |
| *OsTIP1;2* | Os01g0975900 |
| *OsTIP2;1* | Os02g0658100 |
| *OsTIP2;2* | Os06g0336200 |


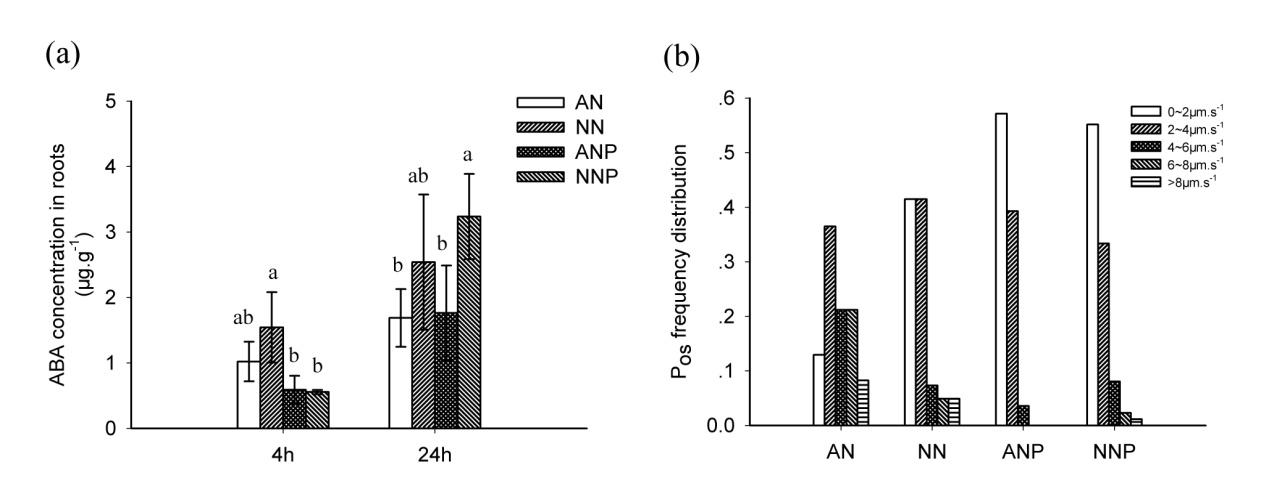


Figure S1 Effects of different nitrogen forms and drought stress on cucumber root ABA concentration (a) and root protoplast water permeability coefficient P_os_ value frequency distribution (b). Cucumber plants were cultured identically to rice plants, and after PEG treatment for 4 h and 24 h, root samples were harvested for ABA detection. The root protoplast isolation and swelling assay was performed according to a previous study (Ding et al., 2015); samples were harvested after 24 h of PEG treatment. The data represent means of three replicates. The error bars indicate the ± SD. Significant differences (P<0.05) between treatments are indicated by different letters. Rice seedlings were supplied with ammonium (AN) or nitrate (NN) under control and drought stress simulated by the addition of 2% PEG6000 (NH_4_^+^ + PEG as ANP; NO_3_^-^ + PEG as NNP).


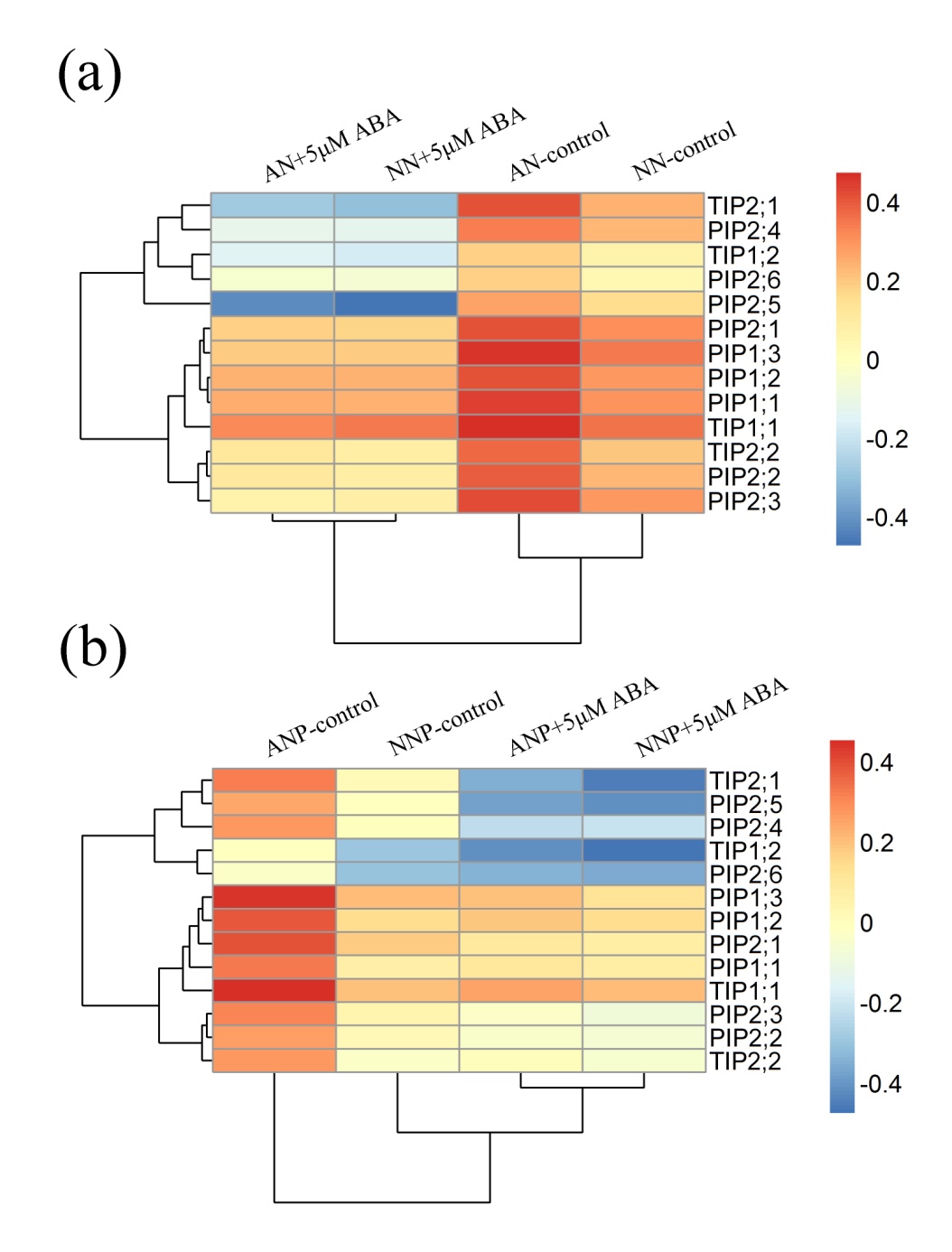


Figure S2 Effects of exogenous ABA on root *PIP* and *TIP* gene expression under non-drought stress (a) and drought stress (b). Exogenous ABA was applied to the nutrient solution at a final concentration of 5μM as soon as drought stress was simulated by using 10% PEG, and after 24 h of treatment, the root samples were harvested for gene expression analysis. The data were analysed as reported in Figure 4. The treatments were ammonium (AN-control) and with ABA (AN + 5μM ABA), nitrate (NN-control) and with ABA (NN + 5μM ABA), ammonium with PEG (ANP-control) and ABA (ANP + 5μM ABA), nitrate with PEG (NNP-control) and ABA (NNP + 5μM ABA).


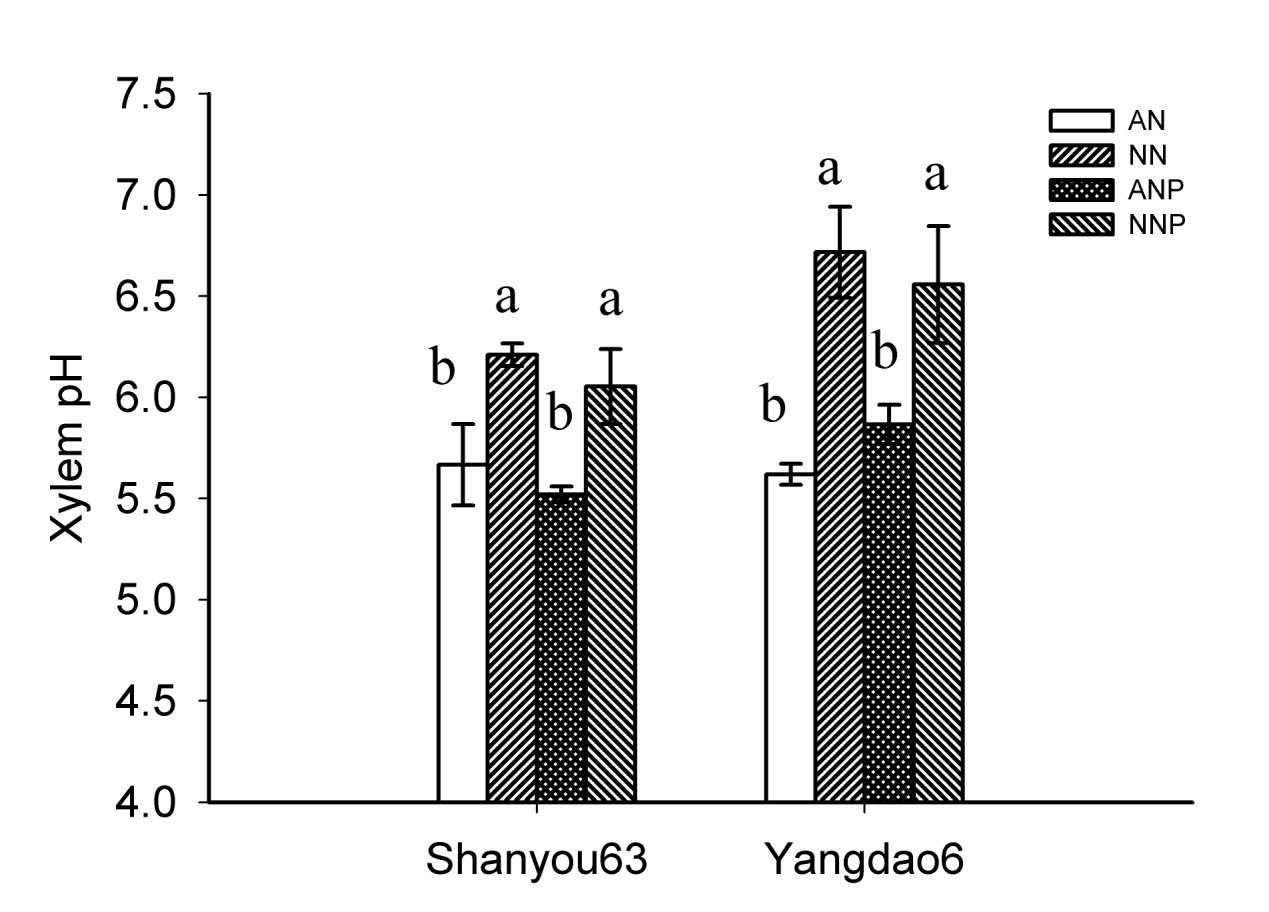


Figure S3 Effects of different nitrogen forms and drought stress on rice plant two cultivars: Shanyou63 and Yangdao6 xylem pH. Xylem samples were harvested and measured using a pH electrode (PB-10, Sartorius) after 10 days of PEG treatment. The data represent the means of three replicates. The error bars indicate the ± SD. Significant differences (P<0.05) between treatments are indicated by different letters. Rice seedlings were supplied with ammonium (AN) or nitrate (NN) under control and drought stress simulation by adding 10% PEG6000 (NH_4_^+^ + PEG as ANP; NO_3_^-^ + PEG as NNP).
